# Calpain activation and progression of inflammatory cycles in Parkinson’s disease

**DOI:** 10.31083/j.fbl2701020

**Published:** 2022-01-13

**Authors:** Andrew Gao, Hannah M. McCoy, Vandana Zaman, Donald C. Shields, Naren L. Banik, Azizul Haque

**Affiliations:** 1 Department of Microbiology and Immunology, Medical University of South Carolina, Charleston, SC 29425, USA; 2 Department of Neurosurgery, Medical University of South Carolina, Charleston, SC 29425, USA; 3 Ralph H. Johnson Veterans Administration Medical Center, Charleston, SC 29401, USA

**Keywords:** *α*-Synuclein (*α*-Syn), Calpain, Dopamine, Neurodegeneration, Neuroinflammation, Spinal cord, Parkinson’s disease (PD)

## Abstract

Parkinson’s disease (PD) is a progressive, neurodegenerative condition of the central nervous system (CNS) affecting 6.3 million people worldwide with no curative treatments. Current therapies aim to mitigate PD’s effects and offer symptomatic relief for patients. Multiple pathways are involved in the pathogenesis of PD, leading to neuroinflammation and the destruction of dopaminergic neurons in the CNS. This review focuses on PD pathology and the role of calpain, a neutral protease, as a regulator of various immune cells such as T-cells, microglia and astrocytes which lead to persistent neuroinflammatory responses and neuronal loss in both the brain and spinal cord (SC). Calpain plays a significant role in the cleavage and aggregation of toxic *α*-synuclein (*α*-syn), a presynaptic neural protein, and other organelles, contributing to mitochondrial dysfunction and oxidative stress. *α*-Syn aggregation results in the formation of Lewy bodies (LB) that further contribute to neuronal damage through lipid bilayer penetration, calcium ion (Ca^2+^) influx, oxidative stress and damage to the blood brain barrier (BBB). Dysfunctional mitochondria destabilize cytosolic Ca^2+^ concentrations, raising intracellular Ca^2+^; this leads to excessive calpain activation and persistent inflammatory responses. *α*-Syn aggregation also results in the disruption of dopamine synthesis through phosphorylation of tyrosine hydroxylase (TH), a key enzyme involved in the conversion of tyrosine to levodopa (L-DOPA), the amino acid precursor to dopamine. Decreased dopamine levels result in altered dopamine receptor (DR) signaling, ultimately activating pro-inflammatory T-cells to further contribute to the inflammatory response. All of these processes, together, result in neuroinflammation, degeneration and ultimately neuronal death seen in PD. 1-Methyl-4-phenyl-1,2,3,6-tetrahydropyridine (MPTP—a prodrug to the neurotoxin 1-methyl-4-phenylpyridinium (MPP+)), rotenone (an environmental neurotoxin), and 6-hydroxydopamine (6-OHDA - a neurotoxic synthetic organic compound) induce PD-like conditions when injected into rodents. All three agents work through similar mechanisms and lead to degeneration of dopaminergic neurons in the substantia nigra (SN) and more recently discovered in motor neurons of the spinal cord (SC). These neurotoxins also increase calpain activity, furthering the neuroinflammatory response. Hence, calpain inhibitors have been posited as potential therapeutics for PD to prevent calpain-related inflammation and neurodegenerative responses in not only the SN but the SC as well.

## 1. Introduction

Parkinson’s disease (PD) is a chronic, progressive neurodegenerative disorder. PD symptoms result from the loss of dopaminergic neurons in the midbrain substantia nigra (SN) pars compacta, culminating in the depletion of dopamine in the striatum [1]. The degenerative changes in PD are not only limited to the nigrostriatal pathway; other sites throughout the nervous system, e.g., the spinal cord (SC), have been shown to also play a critical role in the pathophysiology of the disease [2–4]. Neuron loss manifests symptomatically as tremors, bradykinesia, limb rigidity, and gait/balance abnormalities. The cause of PD is not completely understood, therefore noncurative therapies aim to alleviate symptoms and improve patients’ quality of life [5]. Systemic administration of levodopa (L-DOPA), the amino acid precursor of dopamine, is commonly used for therapeutic treatment of PD [5]. However, L-DOPA only offers temporary relief and can lead to adverse side effects such as motor fluctuations and L-DOPA-induced dyskinesias [6]. Hence, current studies are focused on the identification of potential therapeutic targets for both the SN and SC and development of more effective therapeutic treatments for PD.

It is well known inflammation plays a crucial role in PD’s pathology through destruction of dopaminergic neurons as a result of Lewy body (LB) formation, glial cell activation, and perpetual inflammatory cycles in the SN [1,7–9]. Recently, it has been shown these same mechanisms occur in the SC, destroying motor neurons through abnormal *α*-synuclein (*α*-syn) aggregation and LB formation. LB’s are cytoplasmic, proteinaceous, lipid-rich inclusions composed of *α*-syn aggregates and other biological components such as lipid membrane fragments and distorted organelles [10–12]. LB’s serve as hallmarks of the pathogenesis in PD and other neurodegenerative disorders [10,11]. Studies have shown an association exists between the mechanism driving *α*-syn fibril assembly and the recruitment of other proteins and organelles with the formation of LB [11]. *α-*Syn aggregation may occur as a result of glial cell dysregulation [13].

Glial cells in the central nervous system (CNS) are shown to be activated in PD patients. These glial cells are activated by 1-methyl-4-phenylpyridinium (MPP+), rotenone, and 6-hydroxydopamine (6-OHDA) in both the SN and SC [14,15]. Studies have focused on microglia and astrocytes, the most abundant glia in the CNS responsible for homeostasis maintenance and other metabolic functions [7,16–18]. Upon activation, microglia proliferate and differentiate, altering their number and morphology; they contribute to neuronal death through a variety of mechanisms including inflammation, *α*-syn accumulation, oxidative stress, and damage to the blood brain barrier (BBB) [9,19,20]. Microglial activation forms a positive feedback loop, where dead neurons subsequently activate astrocytes, perpetuating an inflammatory cycle [7,21]. Astrocytes have a variety of functions, some of which are critical for neuronal health including: structural and metabolic support, synaptic transmission regulation, BBB support, production of neurotrophic factors, etc. [22,23]. One such neurotrophic factor is glial-derived neurotrophic factor (GDNF), which is essential for the development and survival of dopaminergic neurons [22]. In healthy individuals, after microglial initiation of an immune response, astrocytes migrate to the damaged area and form a barrier to prevent the spread of toxic compounds [22,23]. In PD patients, however, the function of astrocytes is disrupted, resulting in the degeneration of neighboring neurons in the CNS. It is believed astrocytes may function in a neuroprotective manner in PD due to the effects of astrocyte dysfunction on surrounding neurons in the SN and SC [22,23]. Investigations into the neuroprotective effects of astrocytes need to be conducted to fully understand the capabilities of astrocytes as therapeutic mechanisms.

Other contributors to the inflammatory cycle in PD are T helper (Th) cells, specifically Th1 and Th17 cells [24]. Th cells are activated by various immune substrates and participate in the inflammatory response implicated in the pathogenesis of PD [25–27]. Injured neurons and immune cells in the CNS release various cytokines, for example high mobility group box (HMGB1), that induce T cell differentiation into Th17 cells. Th17 cells are the most inflammatory Th cell phenotype; they exert cytotoxic effects through cytokine and chemokine release [28]. Interleukin-17A (IL-17A), an inflammatory cytokine released by Th17 cells, is cytotoxic to neurons in the CNS, and when released, causes neuronal cell death in the SN and SC [26,29].

Recent studies have also shown PD-related neurotoxins, such as MPTP, activate calpain the SN and SC in animal models [21,30]. Calpain is a neutral protease known to activate glial cells and T-cells in the presence of elevated intracellular calcium (Ca^2+^) concentrations [7]. Calpain activation has been shown to contribute to the disruption of mitochondrial function and dysregulation of various immune cells (i.e., microglia and T-cells). Ca^2+^ activation of calpain also results in the misfolding of *α*-syn, leading to similar dopaminergic neuron loss found in PD patients [31]. *α*-Syn aggregation also causes the disruption of dopamine synthesis through phosphorylation of TH, a critical enzyme in the synthesis of dopamine from L-DOPA [32,33]. Decreased dopamine levels lead to the stimulation of pro-inflammatory immune cells due to the use of high affinity dopamine receptors (DR), DRD3 and DRD5, which are not normally active in healthy individuals [24]. These high affinity receptors induce the Th1 and Th17 cell phenotype, further modulating the inflammatory response promoting dopaminergic neuronal death [24,32]. Similar *α*-Syn aggregation and disruption of motor neurons has also been shown to occur in the SC upon Calpain activation [3]. Therefore, calpain appears to be a critical component of the inflammatory response that results in neuronal cell death in PD patients. Thus, immunomodulatory agents targeting calpain, such as the calpain inhibitor Calpeptin, have been investigated as potential therapies in PD animal models [34,35].

## 2. LB and *α*-SYN aggregation in PD

**Fig. 1.**
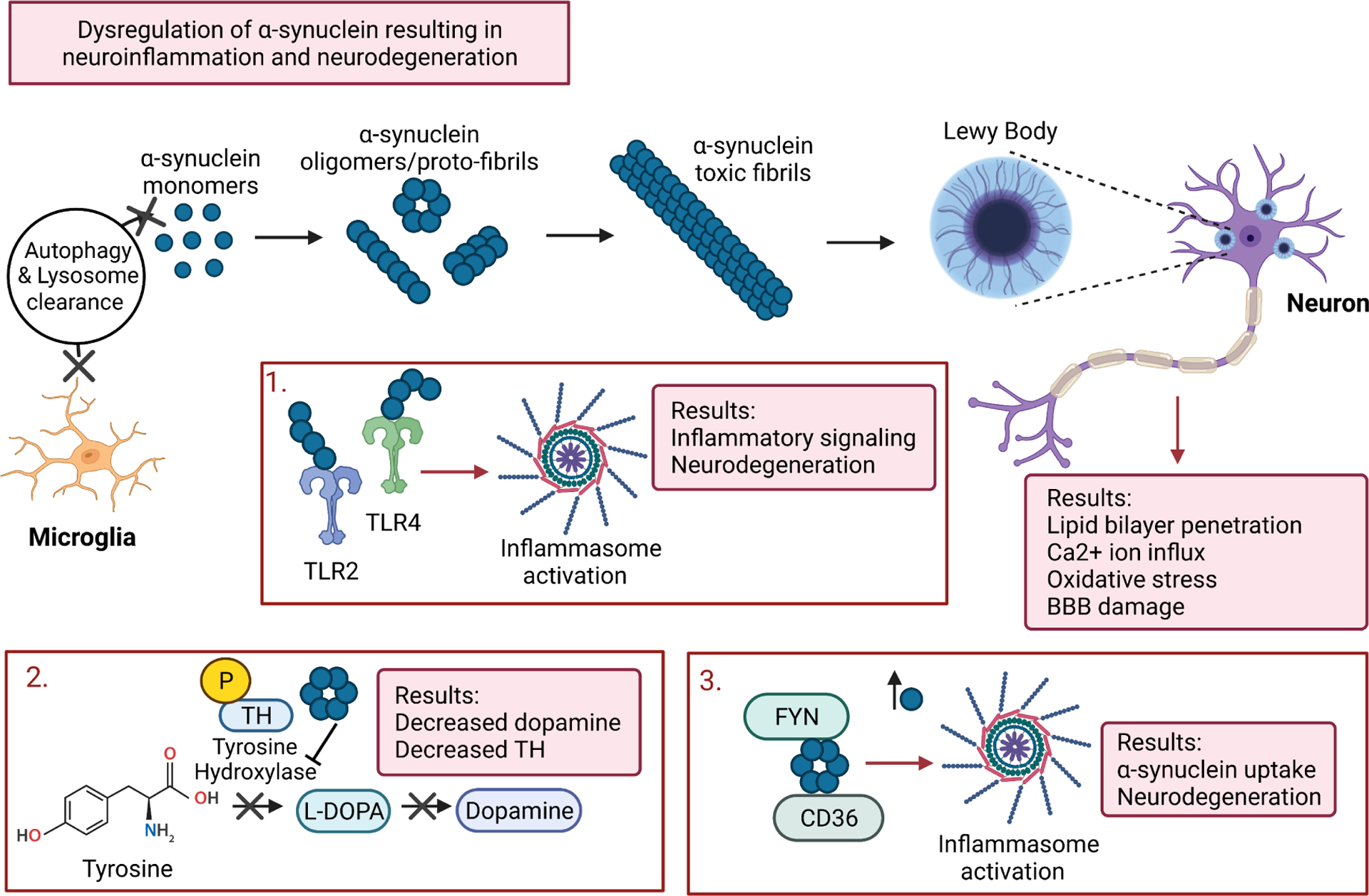
*α*-Syn dysregulation by microglia resulting in neuroinflammation and neuronal death. Impaired microglial function prevents normal autophagy and lysosome clearance, leading to aggregation of *α*-syn monomers into oligomers, proto-fibrils, fibrils, and eventually LB’s. LB, in conjunction with aggregated *α*-syn, results in lipid bilayer penetration, Ca^2+^ ion influx, oxidative stress and damage to the BBB. *α*-Syn aggregation into LB’s results in the activation of TLR2 and TLR4, which further activate inflammasomes leading to inflammatory signaling and neurodegeneration. *α*-Syn aggregation also results in the disruption of dopamine synthesis through the phosphorylation of TH, a critical enzyme in the synthesis of L-DOPA, the amino acid precursor to dopamine. Reduced TH and dopamine levels results in dysfunctional DR signaling leading to further neuroinflammation and neurodegeneration. *α*-Syn aggregation into oligomers and proto-fibrils also allows *α*-syn to interact with CD36 and FYN, resulting in further inflammasome activation, *α*-syn uptake, and neurodegeneration. (Figure created with BioRender.com).

One significant feature of PD is the accumulation of LBs in the CNS [11,36,37]. LBs are presynaptic neural proteins, formed from abnormal clumps of *α*-syn, a neuronal protein implicated in a variety of mechanisms including synaptic vesicle release and recycling, binding of dopamine and serotonin transporters, and synaptic plasticity regulation [38]. LBs themselves are relatively non-toxic; however, oligomerization of *α*-syn forms toxic fibril structures in neuronal plasma membranes (Fig. 1). These toxic fibrils lead to lipid bilayer penetration, pore formation, perturbation of homeostatic Ca^2+^ influx, oxidative stress, and dopamine associated neuronal death in the brain and SC [39]. *α*-Syn concentrations are thought to be controlled by microglia through autophagy and lysosomal clearance. When microglial populations are diminished, however, *α-*syn levels are dysregulated — leading to increased transfer of *α*-syn between grafted neurons and LB formation [40]. Aging, a significant risk factor for PD, is also associated with the decreased ability of microglia to phagocytize *α-*syn [41]. Histological evaluations of neurons taken from postmortem PD samples and MPTP-treated lesioned rats likewise reveal gliosis, neurofibrillary tangles, and LB formation in both the SN and SC [3,21,42].

In healthy individuals, *α*-syn proteins are primarily located in the CNS and comprise 10% of cytosolic protein [43]. In PD patients, however, *α*-syn proteins mutate and truncate, leading to harmful aggregation. The exact mechanisms leading to *α*-syn aggregation are not completely understood, but the process is critical in the progression of PD due to the activation of microglia and disruption of Ca^2+^ homeostasis. Increased levels of misfolded *α*-syn and toxic fibrils allow for significant membrane leakage with neuronal lysis, closely following the release of misfolded and modified *α*-syn proteins into the brain parenchyma [39]. These *α*-syn protofibrils inhibit tyrosine hydroxylase (TH), the rate-limiting enzyme in dopamine synthesis, through TH phosphorylation - ultimately reducing circulating dopamine levels (Fig. 1). Dopamine is believed to be a major regulator of inflammation, involved in the activity, migration, differentiation, and proliferation of immune cells involved in cognitive functions [44]. MPTP-exposed mice express a significant loss of TH neurons with concomitant increase in *α*-syn aggregation and neuronal inclusion formation [7,45]. Semiquantitative analysis of TH-immunoreactive (IR) neurons in sections taken from SN of MPTP injected mice reveals a ~55% reduction of TH-IR dopaminergic neurons compared to controls. Despite the variability in the loss of SN dopaminergic neurons, this MPTP toxin-induced PD model is still widely used to understand the disease’s pathophysiology and to explore the neuroprotective mechanisms.

*α*-Syn aggregation has also been shown to have a negative effect on motor neurons in the SC [3,4,42]. Significant colocalization of TUNEL in neuronal nuclear protein, NeuN, positive neurons highlights neuronal death in both the dorsal and ventral SC [21]. Axonal degradation is also similarly prevalent in MPTP-treated mice, as identified by intense dephosphorylated neurofilament protein (deNFP) IR immunofluorescent staining in the cervical and lumbar SC samples of MPTP-treated mice with phosphorylation of neurofilament proteins (NFP) in these regions [21]. Axonal transport motor proteins are also degraded, with decreasing concentrations of dynein and kinesin found in the dorsal horns (DH), ventral horns (VH), and interneurons (IN) of MPTP-treated mice [7]. Motor protein decay is strongly correlated with PD progression as motor proteins are responsible for synaptic neurotransmission. Studies on the involvement of the SC in PD need to be investigated in order to provide better quality therapeutics to patients suffering from motor function abnormalities and associated pain.

Toll-like receptor 2 and 4 (TLR2 and TLR4) [46], innate immune system proteins upregulated in monocytes and microglia of PD patients, recognize protofibril forms of *α*-syn, prompting the release of pro-inflammatory signals, neuronal degeneration, and activation of inflammasomes (Fig. 1) [38,40]. Additionally, in the *α*-syn driven PD model, Adeno-associated virus(AAV) 2-SY, increased expression of major histocompatibility complex-II (MHC-II) on resident microglia and elevated levels of macrophages and monocytes mediating T cell infiltration is observed compared to controls [47]. Similarly, MHC-I expression on CD8+ T-cells is increased by AAV2-SY transduction, with MHC-I detected outside TH expressing cells. AAV-9-*α*-syn injections in rats also upregulate MHC-I and MHC-II molecules, increasing the presentation of antigenic peptides to cytotoxic T-cells. AAV models, however, are limited by the lack of typical spreading in the CNS by transduced neurons [48].

*α*-Syn also interacts with CD36, an integral membrane protein. Together with an Src family non-receptor tyrosine kinase (FYN), CD36 mediates further *α*-syn uptake and NOD-, LRR- and pyrin domain-containing protein 3 (NLRP3) inflammasome activation, inducing neurodegeneration in various PD models (Fig. 1) [40]. AAV-9-induced expression of *α*-syn results in dopaminergic neuron loss in the SN accompanied by forelimb akinesia, assessed by cylinder tests. Dopaminergic neuron loss is observed in T cell competent rats expressing *α*-syn, but not in T cell-deficient rats [9].

A cross-sectional study conducted for over 20 years on peripheral blood mononuclear cells (PBMCs) stimulated with *α*-syn epitopes revealed the *α*-syn-specific T-cell response is significantly greater in the first ten years prior to PD diagnosis compared to the 17 years following PD diagnosis [49]. Furthermore, the *α*-syn-specific T-cell response is more pronounced immediately following PD diagnosis; this gradually declines afterward [50]. Hence, *α-*syn-specific T cell response screening prior to PD diagnosis may be a viable, early detection paradigm.

## 3. Activated microglia and astrocytes in PD

MPTP and other neurotoxins (rotenone and 6-OHDA) induce inflammatory responses in the brain and SC through activation of microglia and astrocytes in rodent models. Activated microglia and astrocytes release inflammatory factors such as cytokines, chemokines and free radicals that lead to neuronal death (i.e., RANTES, Cox-2, IL-1*β*, IFN-*γ*, and TNF-*α*) [7,14,19]. RANTES, also known as CCL5, and eotaxin chemokine supplementation results in the marked loss of nigral TH-positive neurons in combination with MPTP exposure [51].

Activated microglia also contribute to the inflammatory cycle seen in PD through the recruitment of and interaction with T-cells. Microglia secrete cytokines and chemokines that recruit T-cells to the site of damage, initiating an inflammatory response amplified by incoming immune cells [38]. Activated microglia express increased levels of MHC-I and MHC-II proteins, which are able to interact with CD4+ and CD8+ T-cells, respectively [7]. Both MHC-I and MHC-II house human leukocyte antigen (*HLA*) genes that form complexes with peptide epitopes, presenting intracellular antigens to the CD4+ and CD8+ T-cells [27,49,52]. Once T-cells have migrated to the site of damage, they are able to interact with MHC on microglia and other antigen presenting cells (APCs), leading to further T cell infiltration and consequent inflammatory effects [7].

Microglia are critical components of the inflammatory cycle found in PD pathogenesis due to their ability to be both pro- and anti-inflammatory [53–55]. Microglia are commonly classified with different phenotypes, some being pro- and others anti-inflammatory. Pro-inflammatory microglia present high levels of MHC-II (M1 microglia) and antigens with the capacity to prime T-cells. In contrast, anti-inflammatory microglia express low levels of MHC-II and release beneficial, anti-inflammatory cytokines (M2 microglia). For example, IL-4, a cytokine released by anti-inflammatory microglia, suppresses microglial pro-inflammatory activity and modulates neurogenesis. IL-4 is also implicated in improving neuronal survival and initiating axonal healing following neuronal damage as seen in PD [56]. As PD progresses, pro-inflammatory microglia gradually replace anti-inflammatory microglia, leading to increased inflammation and neurodegeneration. Liposomal clodronate, a potent anti-macrophage agent, attenuates pro-inflammatory macrophage proliferation induced by MPTP-treatment through inhibition of Nuclear Factor Kappa B (NF-*κ*B) phosphorylation [57,58]. Once CD4+ T-cells infiltrate the SN/SC, microglia function as APC’s and present nitrated *α*-syn-derived antigens on MHC-II; this leads CD4+ T-cells to produce interferon gamma (IFN-*γ*) and tumor necrosis factor alpha (TNF-*α*) [24]. Other studies support *α*-syn’s role in upregulating MHC-II on CNS myeloid cells, facilitating the infiltration of IFN-*γ-*producing CD4+ and CD8+ T-cells [52]. These inflammatory markers further contribute to the activation of microglia, and astrocytes.

Glial cells in the ventral-regions of cervical and lumbar SC samples tested for inflammatory markers using immunofluorescent staining confirm MPTP’s role in neurotoxicity [14,59]. Activated microglia (ionized calcium binding adaptor molecule 1 (Iba-1)), astrocytes (Glial Fibrillary Acidic Protein (GFAP)), and peripheral macrophages (ED-2) in the brain and SC samples of MPTP-treated mice support MPTP induced neurotoxicity. Deficiencies of Aquaporin-4 (AQP4), a family of water channel proteins, in MPTP-treated cells exhibit severe PD-like symptoms due to the hyperactivation of microglial pro-inflammatory responses [8]. Other inflammatory markers such as cyclooxygenase-2 (Cox-2), caspase-1, and Nitric Oxide Synthase-2 (NOS-2) are also significantly upregulated in MPTP mouse tissue. Studies showed in comparison to CNS samples harvested from control mice, MPTP-treated mouse tissue show a 39% increase in Cox-2, a 22% increase in caspase-1, and a 112% increase in NOS-2 using Western blotting [21]. In addition, caspase-1 expression causes *α*-syn truncation, triggering additional caspase-1 expression with further inflammation [40]. The increased presence of inflammatory markers suggests neuroinflammation initiated by glial cell activation plays a vital role in the neurodegenerative processes of PD.

## 4. Oxidative stress in PD

The accumulation of *α*-syn in the SN/SC due to microglia depletion has been linked to mitochondrial dysfunction and the generation of reactive oxygen species (ROS) [60,61]. Aggregated *α*-syn contributes to increased oxygen free radical production by interacting with CD11b (an integrin protein found on the surface of innate immune cells) and activation of NADPH Oxidase 2 (NOX2), a crucial enzyme in antimicrobial host defense and immune regulation [40,62,63]. The increase in ROS accumulates in oxidative stress with resulting damage to neurons in the CNS (Fig. 1).

In healthy individuals, ROS and reactive nitrogen species (RNS) levels are maintained through a strict balance of production and clearance [39]. In PD patients, however, ROS and RNS production dramatically exceeds clearance. The unpaired electrons in ROS react with surrounding molecules and mediate the formation of dopamine quinones, resulting in adducts with proteins and other biomolecules which affect structural proteins and enzyme function. For example, dopamine quinones result in the intracellular formation of reactive peroxynitrite in dopaminergic neurons. Peroxynitrite reacts with proteins such as *α-*syn, parkin, DJ-1, and PTEN Induced Kinase 1 (PINK1) through nitratation and nitrosylation of tyrosine and cysteine moieties, respectively. Oxidative stress is then recognized by the ataxia telangiectasia mutated kinase (ATM), and ATM- and Rad3-related (ATR) kinase proteins, resulting in phosphorylation of murine double minute gene 2 (MDM2), a ubiquitin-ligase that enhances p53 degradation [56]. This post-translational modification inactivates MDM2 while simultaneously activating p53. In cases where DNA damage is not corrected, p53 initiates programmed cell death as observed in dopaminergic neurons/PC12 cells [64].

P53-induced pro-apoptotic genes also release cytochrome-c (cyt c), a protein complex linked to oxidative stress, from the mitochondrial membrane. Pyroptosis, a form of programmed cell death induced by the inflammatory caspase cascade, generates a pro-inflammatory milieu with increased levels of IL-1*β* and IL-18 cytokines [52]. Inflammasomes and intracellular multiprotein complexes mediate the maturation of IL-1*β* and IL-18 through caspase-1; these are activated by damage-associated molecular patterns (DAMPs) and pathogen-associated molecular patterns (PAMPs). Activation of inflammasomes and intracellular multiprotein complexes results in a cascade of inflammatory processes contributing to neuronal injury and rapid cell death in both the brain and SC [38,39]. These processes also amplify the ability of innate cells to actively present antigens on MHCs, further directing T cell activation.

## 5. Disruption of blood brain barrier (BBB) in PD

The BBB is compromised by the release of inflammatory factors, i.e., cytokines, following glial cell activation (Fig. 1) [65]. A damaged BBB contributes to dopaminergic neuronal death by facilitating the infiltration of cytotoxic T-cells into the SN [66]. With chronic inflammation, tight junctions between endothelial cells, normally preventing T cell diffusion, allow passage of antibodies and otherwise restricted immune cells [27]. Furthermore, these inflamed CNS endothelial cells upregulate the expression of adhesion molecules, such as intercellular adhesion molecule (ICAM) and vascular cell adhesion molecule-1 (VCAM-1), that bind and recruit circulating T-cells and monocytes. MPTP-induced BBB disruption in mice increases the frequency of CD4+ T-cell infiltration in the ventral midbrain following effector T (Teff) cell transfer [67]. One study suggests retinoic acid receptor (RAR)-related orphan nuclear receptor-*γ*t (ROR*γ*t — a specific transcriptional factor of the Th17 phenotype) positive cells localize around disrupted BBBs in the hippocampus of rats, indicating Th17 cells infiltrate the brain through the BBB [29].

The inflammatory cytokine IL-17A is also correlated with BBB leakage, as IL-17A-KO MPTP-induced mice demonstrate significant alleviation of BBB leakage in the SN [67]. Furthermore, secukinumab, an FDA-approved anti-IL-17 antibody, successfully prevents neuronal death in *ex vivo* cultures [52]. IL-17 activates astrocytes, resulting in the production of multiple chemokines, which in turn act on endothelial cells to disrupt the BBB [29]. Lymphocyte function-associated antigen-1 (LFA-1) is a key T cell integrin, regulating T cell activation and migration [68]. Blocking this LFA-1 activity reduces Treg cell population in SN of MPTP treated mice. Additionally, blocking CD45, a transmembrane molecule, in Tregs impaired the ability of Tregs to protect dopaminergic neurons against MPP+ toxicity [69]. Furthermore, a chronic inflammatory environment is induced by Th17 infiltration through upregulation of the pro-inflammatory and downregulation of the anti-inflammatory microglia phenotype; this promotes local T-cell differentiation into the Th17 phenotype [29].

## 6. Dysregulation of Ca^2+^ homeostasis in PD

**Fig. 2.**
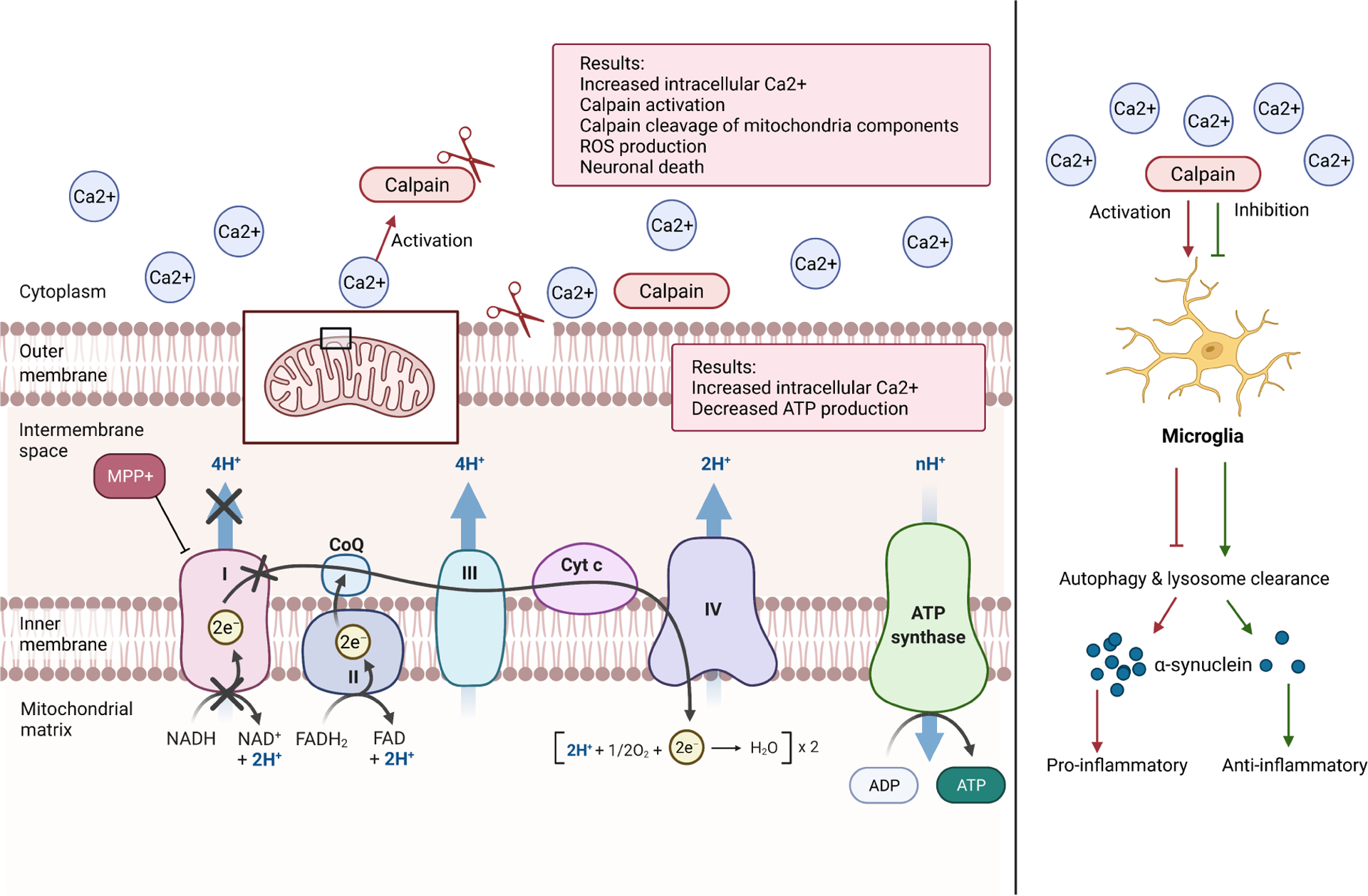
Calpain activation resulting in mitochondrial damage, neuroinflammation, and neurodegeneration. Calpain, a neutral pro-tease, is activated in the presence of elevated intracellular Ca^2+^ levels. Calpain activation leads to the cleavage of various mitochondrial components, resulting in dysfunctional mitochondrial activity. When the ETC is disrupted by calpain, ATP production is reduced. The resulting increase in Ca^2+^ levels causes further calpain activation, ROS production, and neuronal death as a result of microglial activation. Calpain activation results in decreased local microglial populations, disrupting the normal functions of autophagy and lysosome clearance by microglia. Dysfunctional microglia allow for the accumulation and aggregation of *α*-syn, resulting in pro-inflammatory responses. Calpain inhibition, however, allows normal microglial autophagy and lysosome clearance, preventing the accumulation of *α*-syn. (Figure created with BioRender.com).

MPP+, rotenone, and 6-OHDA are mitochondrial toxins which result in: prevention of oxidative phosphorylation, depletion of adenosine triphosphate (ATP), mitochondrial membrane potential disruption, ROS production, and elevation of intracellular Ca^2+^ levels in the brain and SC (Fig. 2) [70]. These neurotoxins raise Ca^2+^ levels by impairing the mitochondrial electron transport chain (ETC), specifically through the inhibition of Complex I. MPTP crosses the BBB and is converted to MPP+ by monoamine oxidase-B (MOA-B), an enzyme localized in CNS astrocytes. Through passive transport, MPP+ enters the mitochondria and inhibits Complex I of the ETC [7,71]. Dysfunctional Complex I depletes cellular ATP levels, leading to a partial depolarization of the cell through reduced Sodium (Na+)/Potassium (K+) ATPase activity. In addition, the overactivation of NMDA receptors results in excitotoxicity, raising cytosolic Ca^2+^ concentrations [65].

Maintaining Ca^2+^ homeostasis is vital for regulating many signaling pathways and biological systems in the body [43]. Among other roles, Ca^2+^ serves as a physiological messenger for transport across the plasma membrane and enzymatic activation [65]. Ca^2+^ is unique from other signaling ions, such as Na+ and K+, as the concentration is 20,000-fold lower in the cytoplasm compared to the extracellular space (significantly less than the ~10- to 30-fold difference for Na+ and K+ ion concentrations). As a result of the extreme concentration gradient, Ca^2+^ acts as a potent intracellular signaling ion, responding rapidly to changes in extracellular and intracellular environments [43]. Thus, controlling Ca^2+^ movement enables a wide range of physiological functions through the activation and inhibition of Ca^2+^-dependent signals.

Irregular Ca^2+^ homeostasis, particularly elevated intracellular Ca^2+^ concentration levels, is implicated in the development of PD through the activation of calpain (Fig. 2), a neutral protease that mediates neuronal death through activation of inflammatory T-cells and microglia [7,72]. Studies showed exposure to MPP+ for over 24 hours causes a significant rise in intracellular Ca^2+^ levels which is four times greater in MPP+ treated hybrid rodent VSC 4.1 motor neuron cells compared to controls [65].

## 7. Calpain activation leading to mitochondrial dysfunction and oxidative stress in PD

MPP+-induced increases in Ca^2+^ significantly upregulate calpain and caspase-3 activity. Calpain activation contributes to the dysfunction of mitochondria by cleaving essential components of the organelle (Fig. 2). For example, *μ*-calpain cleaves the Na^+^/Ca^2+^ exchanger responsible for decreasing cytosolic Ca^2+^ concentrations in normal mitochondria, leading to elevated intracellular Ca^2+^ levels and release of apoptosis-inducing factors [7]. Calpain-10, an atypical calpain, similarly cleaves Complex I of the ETC and ATP Synthase in mitochondria [65]. Other mitochondrial proteins affected by calpain activation include Bcl-2-associated X protein (Bax-2), an apoptotic membrane protein, and B-cell lymphoma 2 (Bcl-2), a survival membrane protein. Bax-2 contributes to apoptotic neuronal death in the brain and SC whereas Bcl-2 protects neurons from cell death. The ratio of Bax-2 to Bcl-2 is significantly increased after calpain activation, mediating apoptosis in neuronal cells. Quantitative analysis of cervical SC samples showed apoptotic Bax proteins are upregulated in MPTP mice [7]. Thus, a cycle is perpetuated where mitochondrial dysfunction leads to calpain activation, which in turn furthers mitochondrial dysfunction with resulting ROS production and ultimate cell death (Fig. 2).

The activation of calpain by MPTP is confirmed in multiple studies. Brain samples of MPTP-treated mice show an MPTP-induced increase in 80 kDa and active 76 kDa forms of m-calpain compared to controls. Furthermore, enhanced formation of 145 kDa calpain-specific spectrin breakdown product (SBDP) is observed in the SN of MPTP-treated mice at a significantly greater level compared to control samples. SBDP is known to colocalize in TH neurons of MPTP-treated mice [65]. Calpain-specific SBDP are associated with acute neuronal damage and act as biomarkers for neurodegenerative diseases such as PD. Thus, a marked increase of SBDP in the SN of MPTP-injected mice suggests calpain plays a crucial role in the progression of dopaminergic neuronal death in PD [7].

## 8. Invasion of inflammatory T-cells in PD

Calpain also influences the infiltration of T-cells as a consequence of calpain induced microglial activation [7]. Pro-inflammatory microglia, activated by calpain induced increase in Ca^2+^, express increased levels of MHC antigens and pro-inflammatory cytokines as opposed to beneficial neurotrophic factors such as Insulin-like growth factor-1 (IGF-1) and GDNF [29,73,74]. Antigenic peptide-loaded MHC molecules, MHC-I and MHC-II, bind to the surface of professional antigen-presenting cells (PAPCs), recognized by either CD4+ or CD8+ T-cells, respectively [43]. CD4+ and CD8+ T-cells, but not B-cells or natural killer (NK) cells, are subsequently activated with subsequent cellular death in PD patients [1]. Some studies, however, demonstrate significantly higher levels of NK cells and B-cells in PD patients, suggesting these cells may also serve as biomarkers of PD [75].

The peripheral population of T-cells is significantly increased in PD patients, supporting T cell trafficking into the CNS [76,77]. Th1 and Th17 cell peripheral blood levels measured in *in vitro* analyses in human PD samples show a significant increase in the frequency of peripheral blood Th1 and Th17 cell concentrations [24]. Although CD8+ T cell concentrations are consistently higher in MPTP-treated mice and PD patients, removal of CD8+ T cell subsets in CD8a−/− mice has not been shown to mitigate MPTP injury. Furthermore, AAV2-SYN treated mice lacking CD8+ T-cells exhibit myeloid inflammatory responses, similar to wild type (WT) mice; thus, CD8+ T-cells may not directly cause the *α*-syn-driven myeloid response. Moreover, Th1 deficiency in mice leads to significant neuroprotection from MPTP insult, suggesting CD4+ T-cells are primarily responsible for mediating the adaptive immune response [1]. AAV2-SYN-treated CD4−/− mice exhibit significantly less activation of myeloid cells, microglia and monocytes, compared to AAV2-SYN-treated WT mice and AAV2-GFP (control)-treated CD4−/− mice. These results suggest CD4+ T-cells are crucial mediators of the pro-inflammatory myeloid response to *α*-syn expression in the SN and SC [47]. Thus, CD4+ T-cells not only drive the activated myeloid response to *α*-syn expression but are also critical to neurodegeneration, as evidenced by decreased TH+ neuronal loss in AAV2-SYN-treated CD4−/− mice.

Infiltration of CD4+ and CD8+ T-cells is significantly elevated in the SN and SC of MPTP-treated mice [21,27]. CD4+ T cell toxicity depends on the Fas/Fas Ligand (FasL) pathway [78]. FasL, a membrane-bound ligand, exhibits significantly decreased affinity for the receptor Fas when mutated. Mice with mutated FasL are characterized by a minor reduction in nigral dopaminergic neurons following MPTP treatment, suggesting CD4+ T-cell-mediated dopaminergic neuronal death relies on functional FasL [1]. In comparison, despite elevated IFN-*γ* levels detected in MPTP-treated mice and post-mortem PD patients, deletion of IFN-*γ* in mice does not significantly alter dopaminergic neuron death. Studies demonstrated mice reconstituted with IFN-*γ*-deficient phenotypes and those lacking CD8+ T-cells are not protected - supporting the theory of T-cell mediated dopaminergic toxicity by CD4+ T-cells and the FasL pathway [79]. Investigations into Calpain’s role as a mediator of the T cell mediated toxicity in PD should be conducted to better understand calpain’s potential as a therapeutic target for PD.

## 9. Anti-inflammatory T-cells neuroprotective role in PD

Treg cells are thought to play an essential role in preventing the onset of PD through migration to the site of injury and interaction with local CNS glia [69]. Activation of glial cells in the CNS causes an inflammatory cascade, ultimately signaling Treg cells to migrate to the SN/SC and perform their immune functions. This process may alter the toxic, reactive microglia phenotype (M1 type) to a non-toxic M2 type. Additionally, the process may drive astrocytes to produce brain-derived neurotrophic factor (BDNF) and GDNF to provide trophic support to SN neurons [80]. Similarly, treatment with granulocyte-macrophage colony-stimulating factor (GM-CSF), such as Sargramostim, before MPTP-intoxication increases Treg cell concentrations in a dose-dependent manner. Diminished neuroinflammatory responses and improved motor function are consequently observed [66,81]. For this reason, Treg cells confer a significant neuroprotective effect via various modes of action including downregulation of pro-inflammatory cytokines, upregulation of anti-inflammatory cytokines, attenuation of Th1-/Th17- driven inflammation, and interaction with local microglia.

In addition, Treg cells provide nigrostriatal protection via cell-cell contact with dopaminergic neurons through the CD47-Signal Regulatory Protein *α* (CD47-SIRP*α*) receptor interaction, triggering Ras-related C3 botulinum toxin substrate 1 (Rac1)/Protein kinase B (Akt) signaling *in vitro* [39]. T-cells harvested from MPTP-treated mice show decreased Treg cell populations compared to control mice (3.6% vs. 1.1%, respectively) [21]. In PD patients, anti-inflammatory Th2 cell phenotypes of CD4+ T-cells, are measured at lower concentrations compared to Th1 cell phenotypes [66]. However, Th1 and Th17 cells demonstrate suppressed pathogenic function after Treg cell administration in PD models [21,69].

Treg cell administration results in reduction of pro-inflammatory cytokines (IL-17, IL-22, IFN-*γ*, TNF-*α*, and IL-1*β*) in the SN of MPTP-treated mice. Studies demonstrate anti-TNF-*α* neutralizing antibodies in combination with Treg cell administration significantly reduce Th1 cell populations [6,82]. Additionally, polyclonal Treg cells activated by anti-CD3 antibodies upregulate anti-inflammatory cytokines, IL-10 and transforming growth factor-*β* (TGF-*β*), in the ventral midbrains of MPTP-treated mice, significantly attenuating neuroinflammation and inhibiting microglial activation [80]. Increases in anti-inflammatory cytokines that mediate inflammatory cell suppression by conversion of adenosine monophosphate (AMP) to adenosine (IL-10, IL-13, Granzyme B (GZMB), and 5’- Nucleotidase Ecto (NT5E)) are observed in cells enriched with Treg markers CD4+, CD25+, and forkhead box P3 (FOXP3+) [77].

Treg cells, specifically CD4+ and CD25+ T-cells expressing Foxp3, function by secreting TGF-*β* and IL-10 to suppress immune cell activation [38]. CD4+ and CD25+ Treg cells harvested from spleen cells of C57BL/6 mice, when activated by anti-CD3 and anti-CD28 antibodies, significantly attenuate dopaminergic neuron loss in comparison to MPTP-treated mice. As a result, dopamine content in the striatum of Treg cell-treated mice is considerably higher [69]. Furthermore, vasoactive intestinal peptide (VIP)-induced Treg cells, from the transfer of pooled splenocytes of N-4YSyn-immunized donors and VIP-treated donors, are able to overcome the toxic environment produced by Th17 amplified N-*α*-syn-mediated nigrostriatal degeneration. *In vitro* data suggests VIP modulated N-4YSyn CD4+ T-cells favor the Treg cell phenotype over the Th17 cell phenotype. Co-cultures of T-cells from N-4YSyn immunized-mice with T-cells from VIP-injected mice demonstrate preferential production of the Treg phenotype rather than that of Th17 [83]. Investigations into Treg cell activity from calpain activation should be conducted to better understand the connection and therapeutic potential.

## 10. Dopamine receptors (DR) and CD4+ T-cells in PD

*α*-Syn is ubiquitously expressed throughout the body; however, as previously discussed, elevated calpain levels have been shown to damage *α*-syn proteins. Harmful *α-*syn aggregation results in LB formation in the SN/SC but also leads to dysfunctional TH enzymes in the CNS with decreased dopamine synthesis [32]. Dopamine is essential for motor control, executive function, motivation and arousal etc. but also modulates the function of immune effector cells through DRs [44,84]. It is hypothesized different DRs may play an important role in the recruitment of *α*-syn associated T cells to the brain through a positive feedback loop during inflammatory responses initiated by calpain activation [85]. *α*-Syn reactive T cells in the periphery, when activated by aggregated *α*-syn, migrate to the CNS due to the disruption of the BBB [33]. BBB disruption allows *α*-syn reactive T cells to infiltrate the CNS and migrate to areas containing high levels of *α*-syn, i.e., the SN and recently discovered in the SC [33]. Once in the SN/SC, T cells interact with *α*-syn and undergo differentiation into various CD4+ T phenotypes; these release various cytokines/chemokines, leading to a cycle of neuroinflammation and neuronal loss [24,33].

**Fig. 3.**
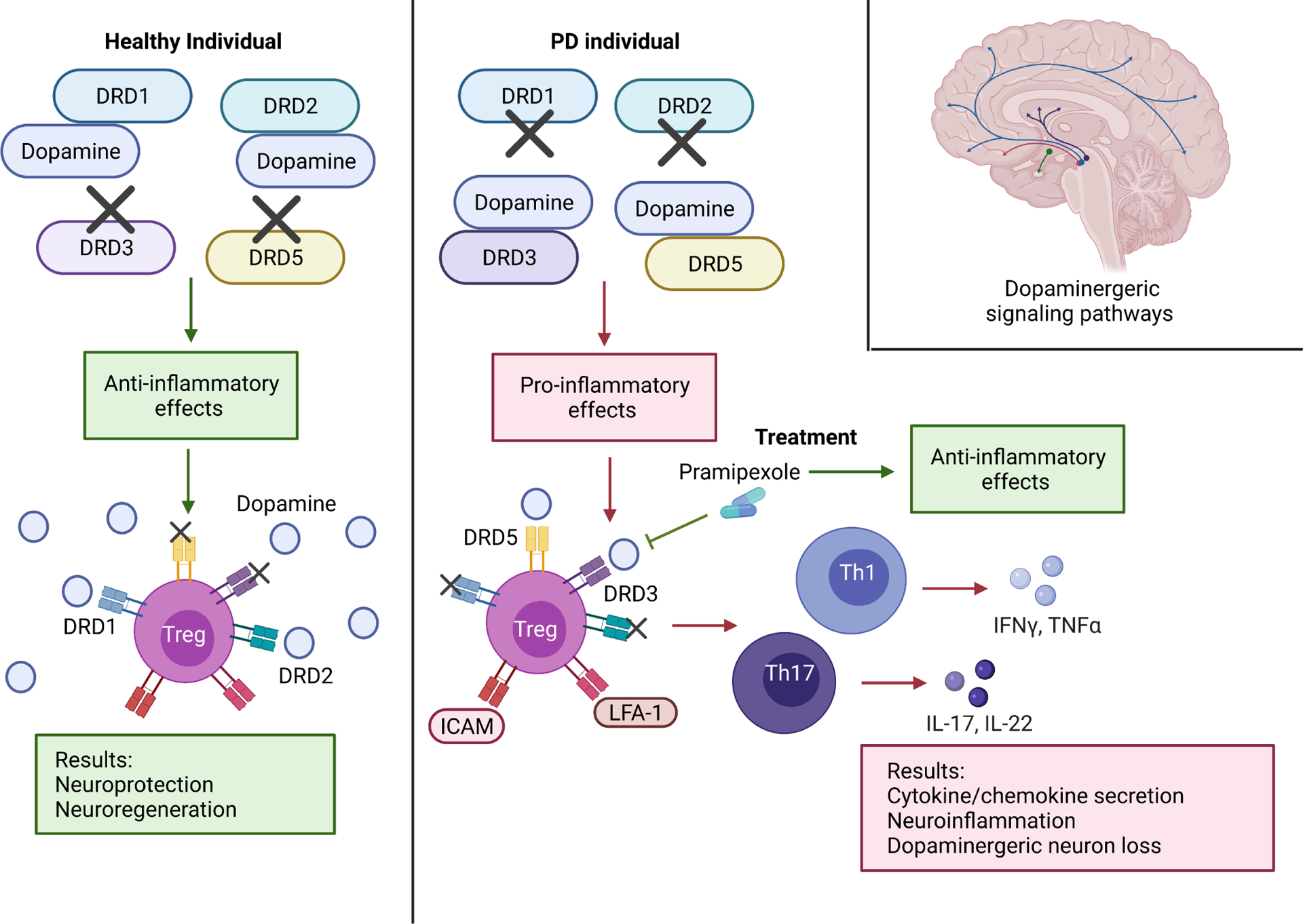
Dysfunctional dopamine signaling resulting in neuroinflammation and dopaminergic neuron loss. In healthy individuals, dopamine signaling is controlled through low affinity DRs (DRD1 and DRD2). DRD1 and DRD2 serve a neuroprotective role and lead to anti-inflammatory responses. In PD individuals, however, decreased dopamine levels prompt activation of high affinity DRs (DRD3 and DRD5) on Treg cells, leading to pro-inflammatory responses. DRD3 and DRD5 on Treg cells interact with dopamine and signal T cell differentiation into Th1 and Th17 cell phenotypes. Treg cell interaction with ICAM and LFA-1 also causes stimulation of the Th17 and Th1 cell phenotypes. These Th cells release pro-inflammatory cytokines and chemokines (IFN-*γ*, TNF-*α*, IL-17, IL-22, etc.), resulting in a pro-inflammatory cascade, neuroinflammation, and dopaminergic neuron loss. Treatment with Pramipexole has been shown to inhibit these high affinity DRs with consequent anti-inflammatory effects. (Figure created with BioRender.com).

Although the expression of different DRs on various CD4+ T cells is poorly understood, it is clear different DRs play an important role in PD pathogenesis. In healthy individuals, dopaminergic signaling relies on low-affinity DRs, DRD1 and DRD2, that exhibit anti-inflammatory effects. Dopamine interaction with these low-affinity receptors decreases cytokine production, regulates inflammasome activity, and suppress inflammation [86,87]. In PD patients, however, the use of high-affinity DRs, such as DRD3 and DRD5, is favored in dopaminergic signaling (Fig. 3). These high-affinity DRs, specifically DRD3, upregulate the migration of inflammatory Th1 and Th17 cell phenotypes [24]. Activation of these receptors on Treg cells also suppresses anti-inflammatory responses.

The Th17 phenotype significantly contributes to dopaminergic neuron death through interaction with LFA-1 and ICAM; Th17 secretion of pro-inflammatory cytokines IL-17, IL-8, IL-21, IL-22, IL-26, TNF-*α*, GM-CSF, and IFN-*γ* also promotes neuronal death [24,67,88]. These cytokines support Th17 cell survival, creating an environment in the brain conducive to chronic neuronal damage [89]. Th17 T-cells thus appear to possess a greater capacity to exacerbate dopaminergic neurodegeneration than Th1 T-cells - identifying the Th17 cell phenotype as the primary driver of neuroinflammation [81].

Furthermore, DRD3 expression favors CD4+ T cell differentiation into the Th1 phenotype rather than the Th17 phenotype [88]. DRD3 is expressed on CD4+ T-cells involved in Th1 differentiation, but not in Treg or Th17 cell differentiation. CD4+ T-cells of DRD3 deficient mice demonstrate significant neuroprotection from MPTP treatment, suggesting the Th1 cell phenotype is a major contributor to neuroinflammation [24]. Likewise, systemic administration of DRD3-antagonists in MPTP-treated mice significantly attenuates nigrostriatal neurodegeneration and motor impairment caused by CD4+ T cell-mediated inflammation (as characterized by microgliosis reduction) [24,32]. Thus, medications blocking the DRD3 receptor, such as pramipexole, demonstrate a neuroprotective effect which may provide clinical benefits in PD patients (Fig. 3) [24]. However, these medications also downregulate dopamine transporter (DAT), a membrane-spanning protein responsible for dopamine reuptake from the synapse, essential for PD patients [88]. Peripheral T-cells in PD patients demonstrate a significant reduction of DAT immunoreactivity, suggesting the peripheral dopaminergic system participates in PD pathogenesis [90].

## 11. Calpain-inhibitors (calpeptin, SJA6017, SNJ-1945) as novel treatment options for PD

### 11.1 Calpeptin

Calpeptin, an inhibitor of calpain, has been evaluated as a potential treatment option in PD animal models [21]. Treatment with calpeptin in MPTP-treated mice leads to a marked reduction in TUNEL and NeuN expression within cervical and lumbar SC samples, providing protection for dorsal and ventral root ganglion neurons [21]. *α*-Syn aggregation is also reduced in both SN and SC of MPTP-treated mice, as demonstrated by immunofluorescent staining using *α*-syn/TH and NeuN antibodies, respectively [7]. In addition, pre-treatment with calpeptin protects SC neurons from MPTP-induced toxicity by preventing neuronal degeneration and axonal alterations, thereby improving gait dynamics and restoring motor functionality in MPTP-treated mice [21].

Bax, an apoptotic protein responsible for neuronal death, is inhibited in the CNS after calpeptin treatment, whereas Bcl-2, is observed in elevated concentrations post-calpeptin treatment. A marked decrease in deNFP IR levels is also detected after calpeptin treatment in the cervical and lumbar SC, demonstrating axonal preservation. Moreover, Treg cell populations exhibit significant growth after calpeptin treatment, resulting in suppression of microglial pro-inflammatory responses and Th1/Th17 cell functions [21].

### 11.2 SJA6017

SJA6017, a cell-permeable calpain inhibitor, is also an effective neuroprotective agent against MPP+ induced damage in spinal motoneurons [65]. This calpain inhibitor provides significant cytoprotection in VSC 4.1 motoneurons after MPP+ induced damage. In addition, SJA6017 attenuates the MPP+ induced rise in intracellular Ca^2+^, reduces SBDP levels, and diminishes ROS elevated after MPP+ exposure. Motor proteins dynein and kinesin also show partial improvement after SJA6017 treatment.

At 5, 10, and 50 *μ*M MPP+ concentrations, pre-treatment with 10 *μ*M SJA6017 effectively increases cell viability and provides resistance to MPP+ induced toxicity [65]. However, at 100 *μ*M and 200 *μ*M MPP+ concentrations, higher concentrations (100 *μ*M) of SJA6017 are required. Pre-treatment with 100 *μ*M of SJA6017 prevents the loss of kinesin and dynein after MPP+ exposure. With ROS production significantly attenuated, cells pre-treated with SJA6017 show a 50% increase in survival rate compared to controls with MPP+ induced toxicity [7]. MPP+ concentrations of 5, 10, and 50 *μ*M also cause a significant increase in calpain and caspase-3 expression compared with controls and SJA6017 pretreated cells. In addition, SJA6017 pre-treated cells show reduced SBDP formation with decreased calpain, pro-caspase-3, and active caspase-3 levels after MPP+ exposure. Resting membrane potentials in VSC 4.1 cells are also reduced after MPP+ exposure compared to controls, but pre-treatment with SJA6017 maintains standard cellular membrane potentials, restoring electrophysiological functionality [65].

### 11.3 SNJ-1945

Calpeptin substantially attenuates MPP+ and rotenone-induced toxicity; however, calpeptin’s application is constrained due to its limited water solubility. Thus, SNJ-1945, a water-soluble calpain inhibitor [91], may serve as a better treatment option for neuroprotection in PD patients. SNJ-1945 pre-treatment alone, at its highest concentration of 250 *μ*M, shows no overt effects on SH-SY5Y-DA and SH-SY5Y-ChAT cells; however, SNJ-1945 is significantly protective at lower concentrations from the effects of MPP+ and rotenone. SNJ-1945 pre-treatment at concentrations of 50, 100 or 250 *μ*M, (dependent on the dosage of MPP+ or rotenone) also attenuates the neurotoxicant-induced inflammatory mediators Cox-2, caspase-1, and p10. Furthermore, calpain activity is diminished after SNJ-1945 pre-treatment, as marked by decreased levels of the 145 kDa calpain-specific SBDP band induced following rotenone and MPP+ exposure [92].

## 12. Conclusions

The role of neuroinflammation in the SN and SC in PD pathogenesis has been elucidated further with recent studies. Calpain, a calcium-activated neutral protease (upregulated after MPTP, rotenone, and 6-OHDA injections) contributes to microglia/astrocyte activation, resulting in the release of various cytokines and chemokines which promote infiltration of toxic T-cells and pro-inflammatory responses. Glial cell activation also causes oxidative stress from ROS/RNS and damage to the BBB, allowing for CNS parenchymal inflammatory progression.

Moreover, calpain plays a significant role in the deletion and cleavage of *α*-syn, which aggregates into harmful LBs with resulting oxidative stress and impaired mitochondrial function. *α*-Syn aggregation also inhibits an essential enzyme in dopamine synthesis (TH), resulting in depleted dopamine levels and altered DR signaling with resulting infiltration of T-cells and ultimately dopaminergic neuron loss.

In addition, calpain activation related to Ca^2+^ homeostasis dysregulation leads to elevated levels of intracellular Ca^2+^, further activating calpain. Thus, calpain inhibitors are being investigated as potential treatment options to prevent neurodegeneration and dopaminergic neuron loss. Calpeptin, SJA6017, and SNJ-1945 are effective calpain inhibitors, mitigating the neuroinflammation and neurodegeneration induced from MPTP, rotenone, and 6-OHDA. These calpain inhibitors effectively decreases calpain levels, reduce inflammation, and protect neuronal viability in both the SN and SC. Further research involving these calpain inhibitors will be required to determine clinical efficacy and safety profiles.
